# 243. Analysis of Risk Factors Associated with Adverse Outcomes Following Calcium Sulfate Bead Use in Periprosthetic Joint Infections

**DOI:** 10.1093/ofid/ofab466.445

**Published:** 2021-12-04

**Authors:** Christine Pham, Holly E Ross, Tara Vijayan, Susan Bukata, Merna Mekheal, Daniela Markovic, Sujan Silakar

**Affiliations:** 1 University of California, Los Angeles; David School of Medicine/University of California, Los Angeles, Los Angeles, California; 2 UCLA Health, Santa Monica, California; 3 UCLA David Geffen School of Medicine, Los Angeles, California; 4 UCLA Health System, Los Angeles, California

## Abstract

**Background:**

Calcium sulfate (CS) beads are increasingly utilized in orthopedic surgeries as a delivery vehicle to administer local antimicrobials intraoperatively. Hypercalcemia, AKI, and elevated serum antimicrobial levels have been reported as potential complications, especially with higher bead volumes. We analyzed the risk factors associated with adverse outcomes among patients with PJIs who received intraoperative CS beads loaded with tobramycin and vancomycin.

**Methods:**

We conducted a retrospective review of adult patients with PJIs who received CS beads from October 2019 to October 2020. Primary outcomes included the incidence of AKI (defined using RIFLE criteria) and hypercalcemia (≥ 11 mg/dL). Logistic regression with forward entry selection of independent variables based on a liberal probability significance of α < 0.25 was used to model the relationships between our variables. Independent variables with clinical relevance that did not meet the conditional selection were also included in the model.

**Results:**

A total of 171 adult patients were included for the analysis. Postoperative AKI occurred in 42 patients (24.6%) who received a mean bead volume of 32 cc. Hypercalcemia occurred in 16 patients (9.4%) who had a mean bead volume of 40 cc. In a univariate analysis, the odds of having AKI and hypercalcemia increased significantly per 10 cc of bead volume with ORs of 1.39 (95%CI, 1.06, 1.82) and 1.65 (95%CI, 1.20, 2.29), respectively. In a multivariate analysis, significant predictors of AKI included: increased bead volume (aOR 1.52; 95%CI, 1.10-2.10), female sex (aOR 2.77; 95%CI, 1.00-7.71), CHF (aOR 3.48; 95%CI, 1.08-11.28), and CAD (aOR 3.90; 95%CI, 1.25-12.18). In the adjusted model, serum tobramycin levels increased (OR 2.67; 95%CI, 1.83-3.90), calcium levels increased with a mean of 0.2 mg/dL (95%CI, 0.12, 0.28), and GFR decreased with a mean of 5.6% (95%CI, 2.8, 8.7) per 10 cc bead volume. In a subset analysis, individuals more likely to experience AKI were patients aged 65 and older (OR 1.9; P=0.039) and had CAD (OR 15.26; P=0.028).

**Conclusion:**

Higher volume of CS beads loaded with vancomycin and tobramycin is associated with adverse outcomes. Older patients with heart disease may be at higher risk for adverse outcomes.

**Disclosures:**

**All Authors**: No reported disclosures

